# Supplementary material for: Robust single-nucleus RNA sequencing reveals depot-specific cell population dynamics in adipose tissue remodeling during obesity
Source: eLife. 2025 Jan 13;13:RP97981. doi: 10.7554/eLife.97981 (PMC11729396; doi:10.7554/eLife.97981)
Supplement: Supplementary file 2. [file elife-97981-supp2.docx]

**Supplementary File 2. Sequences of primers used for quantitative real time PCR (qRT-PCR).**

| Gene | Sequences (Forward) | Sequences (Reverse) |
| --- | --- | --- |
| *Adipoq* | TGTTCCTCTTAATCCTGCCCA | CCAACCTGCACAAGTTCCCTT |
| *Cebpa* | CTGAGAGCTCCTTGGTCAAG | CGAAACCATCCTCTGGGTC |
| *Dlk1* | CTTTCGGCCACAGCACCTAT | CATGGCACCTGCAGACATTG |
| *Emr1* | CTTTGGCTATGGGCTTCCAGTC | GCAAGGAGGACAGAGTTTATCGTG |
| *Fabp4* | AAGGTGAAGAGCATCATAACCCT | TCACGCCTTTCATAACACATTCC |
| *Pecam1* | CTGCCAGTCCGAAAATGGAAC | CTTCATCCACTGGGGCTATC |
| *Plin1* | AATGAGTTGGCCTGCAGAG | AGGCGGGTAGAGATGGTG |
| *Pparg* | GTGCCAGTTTCGATCCGTAGA | GGCCAGCATCGTGTAGATGA |
| *Vcam1* | CTGGGAAGCTGGAACGAAGT | GCCAAACACTTGACCGTGAC |
| *Rplp0* | GAGGAATCAGATGAGGATATGGGA | AAGCAGGCTGACTTGGTTGC |
